# Exploring In Vivo Pulmonary and Splenic Toxicity Profiles of Silicon Quantum Dots in Mice

**DOI:** 10.3390/ma17112778

**Published:** 2024-06-06

**Authors:** Roxana-Elena Cristian, Cornel Balta, Hildegard Herman, Alina Ciceu, Bogdan Trica, Beatrice G. Sbarcea, Eftimie Miutescu, Anca Hermenean, Anca Dinischiotu, Miruna S. Stan

**Affiliations:** 1 Department of Biochemistry and Molecular Biology, Faculty of Biology, University of Bucharest, 91-95 Splaiul Independentei, 050095 Bucharest, Romania; roxana.cristian@drd.unibuc.ro (R.-E.C.); anca.hermenean@gmail.com (A.H.); miruna.stan@bio.unibuc.ro (M.S.S.); 2 DANUBIUS Department, National Institute of Research and Development for Biological Sciences, Splaiul Independentei 296, 060031 Bucharest, Romania; 3 “Aurel Ardelean” Institute of Life Sciences, Vasile Goldis Western University of Arad, 86 Rebreanu, 310414 Arad, Romania; balta.cornel@uvvg.ro (C.B.); herman.hildegard@uvvg.ro (H.H.); alinaciceu80@gmail.com (A.C.); 4 National Institute for Research & Development in Chemistry and Petrochemistry (INCDCP-ICECHIM), 202 Spl. Independentei, 060021 Bucharest, Romania; trica.bogdan@gmail.com; 5 Materials Characterization Department, National Institute for Research & Development in Electrical Engineering (ICPE-CA), 313 Splaiul Unirii, 030138 Bucharest, Romania; gabriela.sbarcea@icpe-ca.ro; 6 Faculty of Medicine, Vasile Goldis Western University of Arad, 86 Rebreanu, 310414 Arad, Romania; miutescu.eftimie@uvvg.ro; 7 Research Institute of the University of Bucharest (ICUB), University of Bucharest, 91-95 Spl. Independentei, 050095 Bucharest, Romania

**Keywords:** silicon quantum dots, mice, pulmonary and splenic toxicity, oxidative stress, histones

## Abstract

Silicon-based quantum dots (SiQDs) represent a special class of nanoparticles due to their low toxicity and easily modifiable surface properties. For this reason, they are used in applications such as bioimaging, fluorescent labeling, drug delivery, protein detection techniques, and tissue engineering despite a serious lack of information on possible in vivo effects. The present study aimed to characterize and evaluate the in vivo toxicity of SiQDs obtained by laser ablation in the lung and spleen of mice. The particles were administered in three different doses (1, 10, and 100 mg QDs/kg of body weight) by intravenous injection into the caudal vein of Swiss mice. After 1, 6, 24, and 72 h, the animals were euthanized, and the lung and spleen tissues were harvested for the evaluation of antioxidant enzyme activity, lipid peroxidation, protein expression, and epigenetic and morphological changes. The obtained results highlighted a low toxicity in pulmonary and splenic tissues for concentrations up to 10 mg SiQDs/kg body, demonstrated by biochemical and histopathological analysis. Therefore, our study brings new experimental evidence on the biocompatibility of this type of QD, suggesting the possibility of expanding research on the biomedical applications of SiQDs.

## 1. Introduction

Semiconductor nanocrystals or quantum dots (QDs) are inorganic nanostructured nanomaterials with unique optical and spectroscopic properties containing metals, such as cadmium, which can form compounds with selenium or tellurium, resulting in CdSe or CdTe. These crystals can bind long-chain alkyl-thiol compounds on their surface, being used in the last decade as fluorescent compounds [1,2]. Compared to organic fluorophores, QDs show unique properties, with the most important being quantum confinement. Therefore, the structural modification of QDs contributes to the change in their optical properties. 

QDs present high photostability towards organic dyes but have low biocompatibility caused by the presence of heavy metals in their structure. Nanoparticle encapsulation represents a solution to obtain superior particles regarding structural stability and quantum yield. Long-term exposure of the particles to environmental factors, such as blue light or UV, leads to the quenching of QD fluorescence. In this context, adding a protective coating has proven effective in increasing the resistance of QDs to photobleaching [3,4]. It was also noted that the presence of an additional encapsulation layer reduced the toxicity of the QDs, which contributed to the expansion of fields of applicability [5]. 

Currently, the general aim is to increase the biocompatibility of QDs by substituting heavy metals in the structure with silicon. Silicon QDs (SiQDs) are considered less harmful than those containing heavy metals [6,7,8] and can be used in medical applications such as bioimaging, fluorescent labeling, drug delivery, protein detection techniques, and tissue engineering [9]. 

The methods of QD synthesis were grouped into two large categories: the “top-down” approach, with the largest problem of obtaining an imperfect surface structure; and the “bottom-up” approach, which creates less waste and is more economical, and it can be divided into two broad categories—chemical and physical [10]. Laser ablation is included in the physical methods and is considered the cleanest method for the synthesis of QDs because it produces the least amount of waste [11]. This method has the advantage that it does not require chemical precursors and ensures sterility; the experimental conditions are easy to obtain, not requiring high temperatures or very high pressures [12].

SiQDs can enter the body through the skin and respiratory or digestive tracts. Once in the body, the way they interact with cells is influenced by their size, shape, chemical structure, and surface properties [13]. These characteristics also influence other aspects, such as the accumulation/excretion ratio of the particles, the level of cytotoxicity [14,15], and the type of their cellular internalization, which can be represented by phagocytosis, macropinocytosis, clathrin- or caveolin-mediated endocytosis, and non-clathrin- or non-caveolin-mediated endocytosis [16]. Once inside the body, QDs are transported through the blood circulation and distributed mainly in the lungs, spleen, liver, and kidneys, but also in the reproductive system and brain, as they can cross the blood–brain barrier, being able to generate cytotoxic and genotoxic effects [17].

Previously, the exposure of Sprague Dawley rats to Cd/Se-ZnS QDs functionalized with amino or carboxyl terminal groups induced lung damage and inflammatory processes, depending on injected dose and exposure time [18]. The potential of SiQDs to trigger an inflammatory reaction was also evaluated in vitro on MRC-5 human lung fibroblasts, revealing their impact on membrane integrity. This was indicated by the gradual increase in lactate dehydrogenase levels over time, along with the accumulation of autophagosomes and lysosomes. Inflammatory processes were observed at the cellular level, evidenced by increased levels of nitric oxide and interleukin-6 and the appearance of redox imbalances in the turnover of the extracellular matrix [19], most probably due to an increase in reactive oxygen species (ROS) [20]. However, the research on near-infrared QDs intravenously administered to BALB/C mice at the necessary dosage for in vivo tumor imaging indicated no evidence of toxicity in the lungs and spleen [21].

Taking into consideration all these controversial results, it is necessary to carry out more in-depth studies to decipher the molecular mechanisms induced by the presence of SiQDs. In this way, our present research aimed to conduct an in vivo toxicological assessment of SiQDs by examining the extent of oxidative stress linked to alterations in morphology and the inflammatory state in the lungs and spleen of mice.

The lungs and spleen were selected for evaluating SiQD toxicity, considering their roles as potential accumulation sites and clearance organs. While intravenous administration bypasses direct lung exposure, the lungs remain relevant due to their status as a potential secondary deposition site following systemic circulation. SiQDs can potentially accumulate in lung tissue via extravasation from the bloodstream or uptake by pulmonary macrophages. Additionally, the spleen’s involvement is crucial given its function in filtering blood-borne particles and its high density of macrophages, which may sequester SiQDs.

## 2. Materials and Methods

### 2.1. Synthesis of SiQDs and Their Characteristics

**Figure 1:**
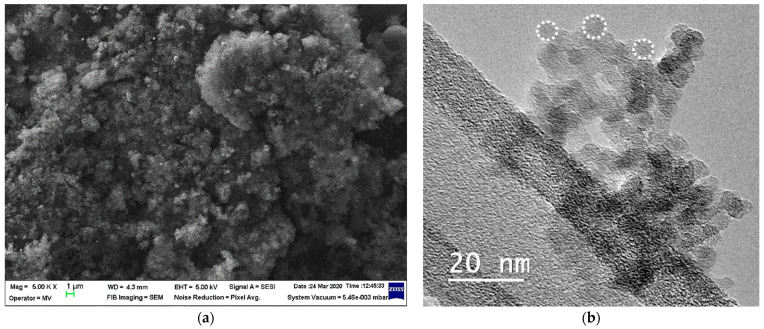
The characterization of SiQDs was achieved by SEM (**a**) and TEM (**b**) analysis. The spherical shape of QDs is marked by white dot circles (**b**).

QDs purchased from the National Institute of Laser, Plasma and Radiation Physics, Bucharest, Magurele, Romania, were used to carry out this study. For the synthesis of QDs, the technique detailed by Grigoriu et al. [22] was used. This method involved ablation in a stainless-steel chamber, with a laser beam concentrated on a silicon target. The collected particles reacted further with oxygen in the air, which provided a silicon dioxide layer on their surface, resulting in core/shell QDs of 6 to 8 nm composed of silicon/silica. The QDs displayed a high tendency of aggregation as shown by the scanning electron microscopy (SEM) images in Figure 1a, with clusters of QDs being noticed. However, their average sizes of less than 10 nm were evidenced in the transmission electron microscopy (TEM) images (Figure 1b).

### 2.2. Administration of SiQDs to Mice

To carry out this study, adult male Swiss mice were used, each weighing approximately 25 g. The mice were housed in the Animal facility of “Vasile Goldiș” Western University of Arad in compliance with the European Union and national regulations (Ethical Committee Approval no. 14 from 27 October 2015 of “Vasile Goldiș” Western University of Arad). Mice received a standard diet of pellets and water, were maintained at 20–25 °C, and were subjected to a circadian rhythm of 12 h light and 12 h dark. These conditions remained constant throughout the experiment. Afterward, the mice were randomly divided into four batches, with each corresponding to specific time intervals (1 h, 6 h, 24 h, and 72 h). Within each of these, there were four groups, each consisting of 5 mice: a control group that received saline, and the other groups that received doses of 1 mg QDs/kg b.w., 10 mg QDs/kg b.w., and 100 mg QDs/kg b.w. Treatments were administered by the intravenous injection of 200 µL of each dose in the tail vein. At 1 h, 6 h, 24 h, and 72 h post injection of the QDs, all mice were euthanized, and the organs selected for this study (lungs and spleen) were collected for biochemical and histological analyses.

### 2.3. Histology and Immunohistochemistry

Tissue sections from both the lung and spleen were first fixed using a 4% formaldehyde solution in phosphate-buffered saline, and then enclosed in paraffin. Subsequently, these sections underwent the conventional hematoxylin and eosin staining procedure. For microscopic evaluations, an Olympus BX43 microscope with an attached Olympus XC30 digital camera (Olympus, Hamburg, Germany) was utilized to examine the stained slides.

### 2.4. Obtaining the Tissue Homogenates

Tissue homogenization was performed in a ratio of 0.1 mg of tissue to 1 mL of 0.1 M TRIS-HCl–5 mM EDTA buffer (pH 7.4). The homogenization process was performed thrice, with each session lasting two minutes, using a mixing mill (Retsch MM301, Retsch GmbH, Haan, Germany) at 30 vibrations/second frequency. The resulting suspension was centrifuged at 10,000× *g* for 10 min at 4 °C, and the supernatant was carefully collected and stored at −80 °C. Total protein content was determined using the method based on the Folin reagent and bovine serum albumin as a standard.

### 2.5. Measurement of Antioxidant Enzyme Activities

To measure the activity of antioxidant enzymes, the following techniques were used. The method described by Aebi [23] was used to determine catalase activity (CAT), based on the reduction in H_2_O_2_ concentration at 240 nm. Superoxide dismutase (SOD) activity was assessed based on the oxidation of NADH, leading to a decrease in absorbance at 340 nm [24]. Using GSH and tert-butyl hydroperoxide as substrates, glutathione peroxidase (GPx) activity was measured at 340 nm [25]. For the glutathione reductase (Gred) activity, changes in absorbance intensity at 340 nm were monitored following the conversion of NADPH to NADP^+^, a consequence of the reduction of GSSG to GSH [26]. In addition, the rate of conjugation of 1-chloro-2,4-dinitrobenzene (CDNB) to GSH was used to assess glutathione S-transferase (GST) activity [27]. Absorbance intensity was measured using a JASCO V-530 spectrophotometer (Jasco, Tokyo, Japan). To express enzyme activities, they were normalized by protein concentration, presented as activity units per milligram of protein (U/mg) and as a percentage of control.

### 2.6. Determination of Malondialdehyde Level (MDA)

The level of MDA was assessed using a reference solution of 1,1,3,3-tetramethoxypropane. Initially, a volume of 700 µL of 0.1 M HCL was used and was added to both the MDA calibration curve and the cell lysates. The mix was maintained at room temperature for 20 min. After this, a volume of 900 μL of thiobarbituric acid (TBA; 0.025 M) was pipetted into all tubes, and the samples were incubated for 65 min at 37 °C to facilitate the formation of TBA-MDA products. Subsequently, fluorescence intensity was measured using a Jasco FP-6300 fluorimeter (Jasco, Tokyo, Japan) and converted to nmol MDA based on the reference curve.

### 2.7. Determination Reduced Glutathione (GSH) Level

The glutathione test kit from Sigma-Aldrich (Burlington, MA, USA) was used to determine the level of GSH, following the protocol provided by the manufacturer. This method involves the reduction of 5,5′-dithiobis-2-nitrobenzoic acid (DTNB) by GSH, leading to 5-thio-2-nitrobenzoic acid. Cell extracts were appropriately diluted and deproteinized using 5% 5-sulfosalicylic acid (SSA). Subsequently, centrifugation at 3000× *g* for 5 min at 4 °C was performed to remove precipitated proteins. A volume of 10 µL of the supernatant was mixed with 150 µL of buffer solution containing DTNB (1.5 mg/mL). Following the reaction, a yellow product was obtained, whose absorbance was measured at 412 nm on the Tecan Genious spectrophotometer (Tecan, Männedorf, Switzerland). Finally, an extrapolation was made using a GSH standard curve, and the data obtained are expressed in nmol/mg protein.

### 2.8. Western Blot

The p53, Beclin-1, LC-3, and Nrf-2 protein levels were assessed in samples collected from the control group and mice that received 100 mg SiQD/kg b.w. and were sacrificed at 1, 6, 24, and 72 h. Cell lysates corresponding to 100 µg of protein were run on 10% SDS-PAGE under reducing conditions and then transferred on a 0.4 µm polyvinylidene difluoride (PVDF) membrane in a wet transfer tank (Bio-Rad, Hercules, CA, USA). Subsequently, the membranes were blocked using the blocking solution in the WesternBreeze Chromogenic kit (Invitrogen, Rockford, IL, USA) for 30 min at room temperature. The detection of p53, LC-3, and Nrf-2 proteins was performed with rabbit polyclonal anti-p53, anti-LC-3, and anti-Nrf-2 primary antibodies, respectively (at a 1:250 dilution). The detection of Beclin-1 protein involved the anti-Beclin-1 mouse polyclonal primary antibody (1:250 dilution, SantaCruz Biotechnology, Dallas, TX, USA). Following the incubation with primary antibodies, the membranes were processed using the manufacturer’s instructions, employing alkaline phosphatase-conjugated anti-mouse and anti-rabbit secondary antibodies, along with 5-bromo-4-chloro-3′-indolephosphate/nitroblue tetrazolium as the chromogenic substrate. The resulting bands were visualized and captured using a transilluminator (ChemiDoc MP Video Documentation System, Bio-Rad, Hercules, CA, USA) and subsequently analyzed using the GelQuant.NET software version 1.7.8.

### 2.9. Detection of 8-Hyroxy-2′-deoxyguanosine (8-OHdG)

The evaluation of nucleic acid damage as a result of oxidative stress induced by nanoparticles commonly employs 8-OHdG as a widely recognized biomarker [28]. The relative level of 8-OHdG was determined in murine lung and spleen tissues collected at 72 h after the administration of SiQDs (100 mg/kg b.w.) using an ELISA kit (Abcam, Cambridge, UK). Tissue samples from five individuals within the same group were combined for the analysis. This technique, previously utilized in molecular biology assessments, reduces inter-individual variations and provides an overall perspective of the analyzed group [29,30]. DNA extraction was conducted utilizing the PureLink^®^ Genomic DNA Kits from Invitrogen.

### 2.10. Quantification of 5-Methyl Cytosine (5-mC)

The global level of DNA methylation, as determined by the ELISA technique, was evaluated in murine lung and spleen tissue samples collected 72 h after the administration of 100 mg SiQDs/kg b.w. The assessment was facilitated using the 5-mC DNA ELISA Kit from Enzo Sciences (Farmingdale, NY, USA). This methodology used the previously mentioned extracts to detect 8-OHdG, adjusting the volumes according to the kit’s instructions.

### 2.11. Assessment of Histone Methylation

The initial step involved the extraction of total histones using the EpiQuik™ Total Histone Extraction Kit (Epigentek, Farmingdale, NY, USA). Subsequently, the protein concentration was determined using the Bradford method, followed by detecting histone H4 modifications (methylation, acetylation, and phosphorylation) using the EpiQuik™ Histone H4 Modification Multiplex Assay Kit (Epigentek, Farmingdale, NY, USA).

### 2.12. Statistical Analysis

Statistical analysis for all tests was conducted using GraphPad Prism Version 9 (GraphPad Software, Inc., La Jolla, CA, USA) by one-way ANOVA followed by Tukey’s post hoc multiple comparison test against the control. The results are presented as the mean value ± standard deviation (SD) (*n* = 3). A *p*-value less than 0.05 was considered statistically significant.

## 3. Results

### 3.1. Histopathology of the Lungs and Spleen of Mice Exposed to SiQDs

**Figure 2:**
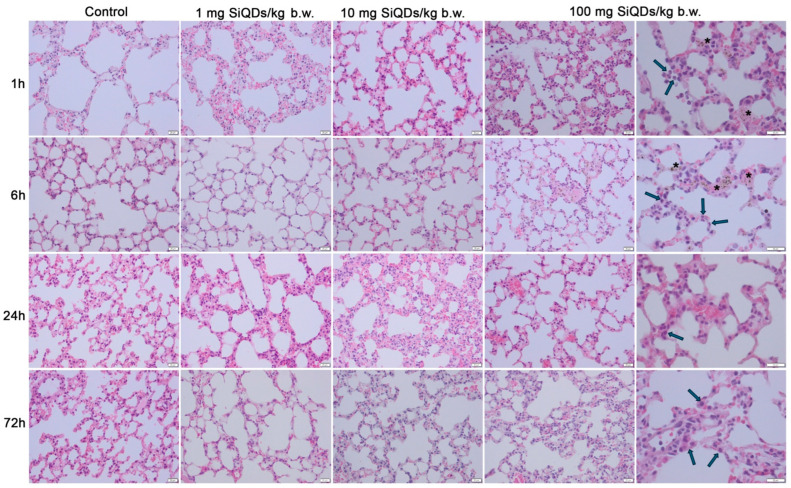
Histopathological evaluation of lung tissue using hematoxylin and eosin staining at intervals of 1, 6, 24, and 72 h post administration of 1 mg, 10 mg, and 100 mg of SiQDs/kg of b.w. Legend: arrows—alveolar macrophages; black stars—SiQD accumulation (brown). Scale bar (represented in the lower right corner of each image): 20 µm.

Examination via light microscopy of the pulmonary tissue shows inflammation characterized by a marked increase in neutrophils (Figure 2—arrows) at 1 h post administration of the highest dose (100 mg SiQDs/kg b.w.), with a reduction observed at 72 h as alveolar macrophages become more prevalent (Figure 2—arrows). For the other two doses, the microscopic structure of the lung tissue remained comparable to that of the control group. QDs (observed as brown dots in Figure 2—stars) were detected in the tissue exclusively up to 6 h post injection when the dose administered was 100 mg QDs/kg b.w.

**Figure 3:**
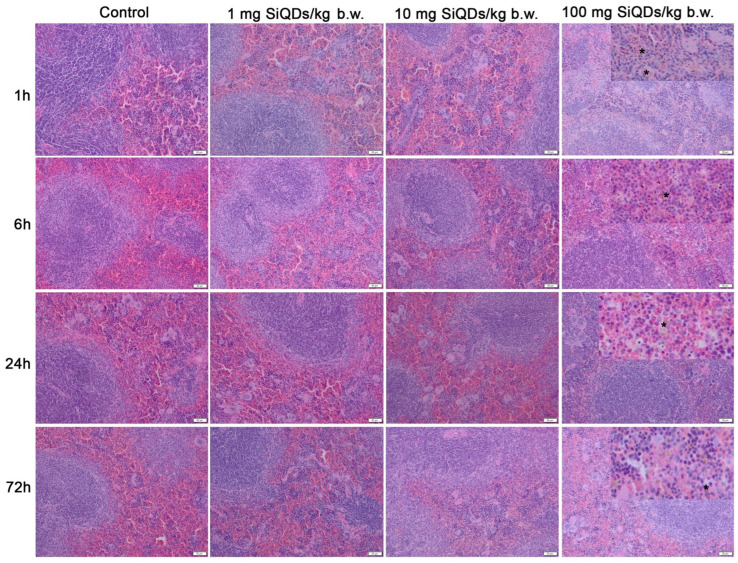
Histopathological evaluation of spleen tissue using hematoxylin and eosin staining at intervals of 1, 6, 24, and 72 h post administration of 1 mg, 10 mg, and 100 mg of SiQDs/kg of b.w. Details for 100 mg QDs/kg b.w. show the SiQD accumulation (brown dots evidenced by stars). Scale bar: 50 µm.

The microscopic structure of the spleen’s white and red pulp in mice remained largely unchanged following treatments with SiQDs. Despite this, the QDs were clearly present in the tissue at all time points examined (brown dots shown in Figure 3—stars).

### 3.2. Analysis of Oxidative Stress Induced by SiQD Administration

**Figure 4:**
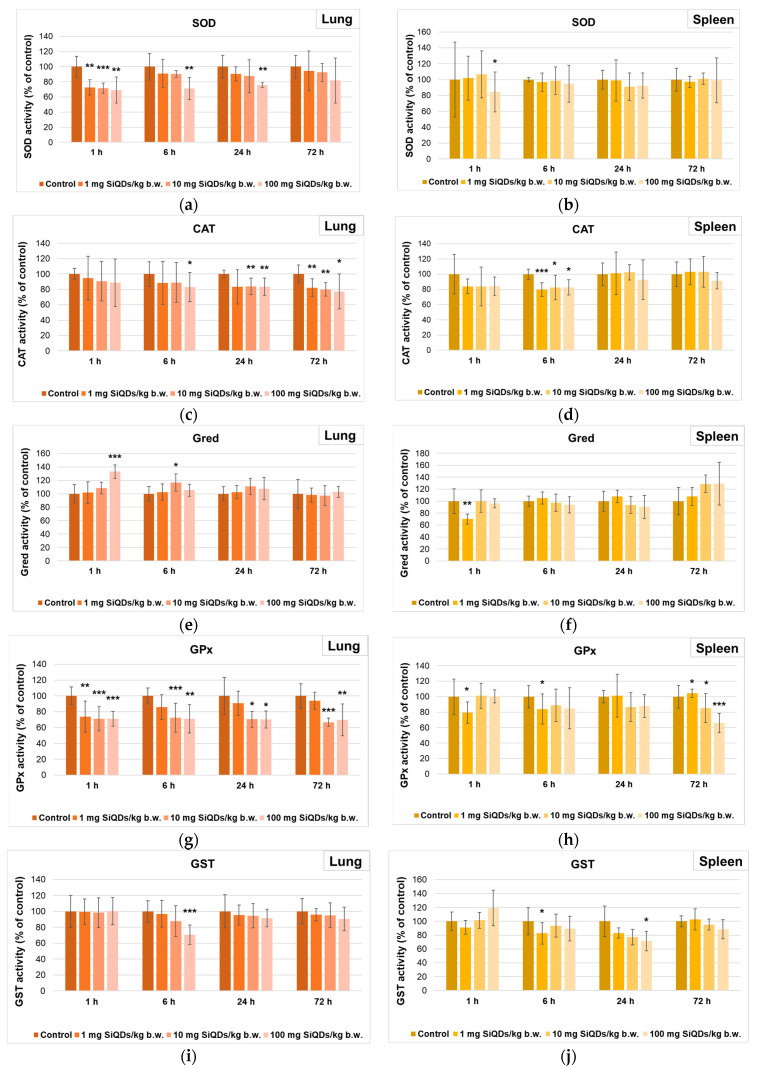
Specific activities of SOD (**a**,**b**), CAT (**c**,**d**), Gred (**e**,**f**), GPx (**g**,**h**), and GST (**i**,**j**) in lung (**a**,**c**,**e**,**g**,**i**) and spleen (**b**,**d**,**f**,**h**,**j**) tissues collected at 1, 6, 24, and 72 h after SiQD administration. Results are calculated as mean ± SD (*n* = 5) and are represented relative to the control. * *p* < 0.05, ** *p* < 0.01, and *** *p* < 0.001 compared to control.

The measurement of enzyme activity in the lung samples indicated a sudden decrease in the SOD activity (Figure 4a), by 31% after 1 h from the administration of 100 mg SiQDs/kg b.w. compared to the control, indicating a superior sensitivity of the lungs to the action of SiQDs, as this decrease was maintained up to 24 h. In the case of splenic tissue (Figure 4b), the lack of generation of superoxide anions in mouse splenic cells was noted based on the fact that SOD activity was almost similar to the control, indicating the ability of the spleen to neutralize the toxic effects produced by SiQDs.

As shown in Figure 4c, the level of CAT activity in the lungs of mice that received SiQDs underwent a reduction compared to the control during the whole experiment, and the CAT activity decreased by 23% relative to the control in the 72 h samples of the 100 mg QDs/kg b.w. group, indicating the presence of oxidative stress, because the cellular defense mechanism was affected. In the spleen samples (Figure 4d), an initial decrease in CAT activity was observed after 1 h, with the lowest level being recorded after 6 h from the administration of 1 mg SiQDs/kg b.w. (by 20% lower compared to the control level). Further, the recovery of this enzyme activity towards values close to those of the control was noticed after 24 and 72 h, suggesting the ability of the spleen to neutralize the toxic effects induced by the presence of SiQDs in this tissue.

Figure 4e shows an increase in Gred activity in the lung tissue, with the maximum value being recorded for 100 mg SiQD/kg b.w. samples, being 33% higher than that in the control after 1 h. After 72 h, the Gred activity returned to values close to the control activity. In the case of splenic tissue samples (Figure 4f), an increase in this enzyme activity was observed after 72 h, with the maximum value being recorded as 29% higher than that of the control for 100 mg SiQDs/kg b.w. samples.

The decrease in GPx activity was more pronounced in lung tissue during the first 24 h post injection compared to that in the spleen. After 72 h, for 100 mg SiQD/kg b.w. samples, almost the same value of this enzyme activity was noticed (70% and 66% of the control in the lung (Figure 4g) and spleen (Figure 4h), respectively).

Regarding the GST activity in the lung samples, a dose-dependent decrease was observed after 6 h, being diminished by 30% compared to the control in the case of 100 mg SiQDs/kg b.w. (Figure 4i). After 24 h and 72 h, the GST activity returned to values closer to the control ones. In the case of spleen samples, a dose-dependent decrease in GST activity was noted after 24 h, being at 72% of the control in the case of 100 mg SiQD/kg b.w. samples (Figure 4j).

**Figure 5:**
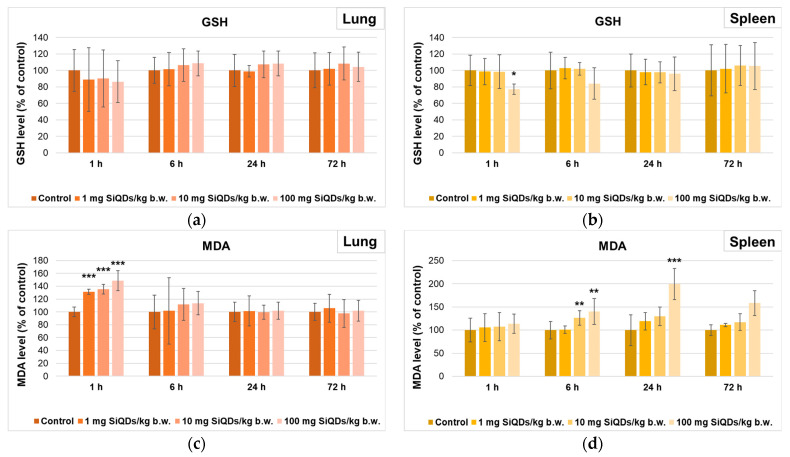
Levels of GSH (**a**,**b**) and MDA (**c**,**d**) in lung (**a**,**c**) and spleen (**b**,**d**) tissues collected at 1, 6, 24, and 72 h after SiQD administration. Results are calculated as mean ± SD (*n* = 5) and are represented relative to the control. * *p* < 0.05, ** *p* < 0.01, and *** *p* < 0.001 compared to control.

Regarding the level of GSH, there was a slight decrease compared to the control after 1 h in the lungs of mice that received 100 mg SiQDs/kg b.w. (Figure 5a), being further increased after 6 h. In the case of the spleen, the concentration of this antioxidant recorded a decrease after 1 and 6 h compared to the control (Figure 5b). However, the GSH level returned to values close to those of the control after 72 h in both types of tissues, suggesting the antioxidant mechanism’s recovery at the cellular level.

The measurement of MDA level as an indicator of lipid peroxidation evidenced a dose-dependent increase in the lung after 1 h (being 49% higher than in the control for 100 mg SiQDs/kg b.w.), but after 24 and 72 h, the level was similar to that of the control (Figure 5c). In contrast, the MDA level in the spleen increased in a dose- and time-dependent manner, being double compared to the control after 24 h in the case of 100 mg SiQDs/kg b.w samples (Figure 5d).

### 3.3. Analysis of Proteins’ Expression Involved in Antioxidant Defense System, Apoptosis, and Autophagy

**Figure 6:**
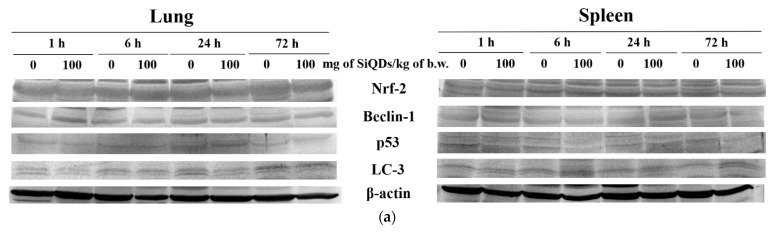

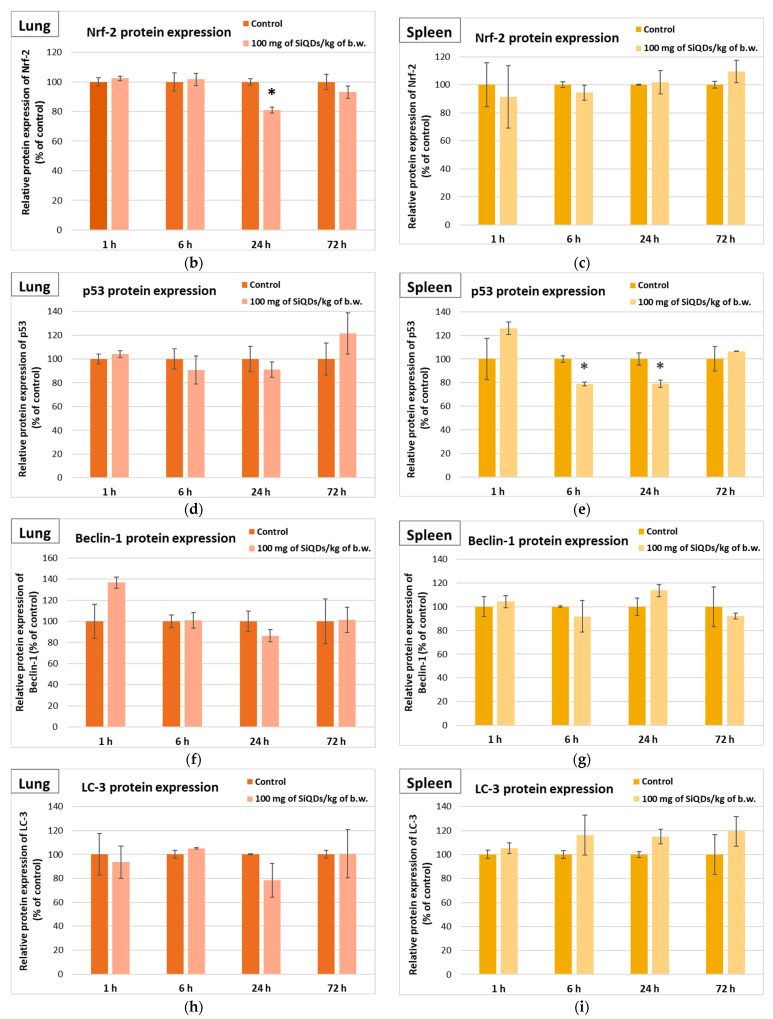
Changes in proteins’ expression involved in antioxidative defense response, apoptosis, and autophagy after SiQD administration (100 mg/kg b.w.) to mice. The analysis of Nrf-2, p53 Beclin-1, and LC-3 protein expression by Western blot (**a**) was quantified in the lung (**b**,**d**,**f**,**h**) and spleen (**c**,**e**,**g**,**i**) tissues collected at 1, 6, 24, and 72 h after SiQD administration. Results are calculated as mean ± SD (*n* = 5) and are represented relative to the control. * *p* < 0.05 compared with the control.

The measurement of Nrf-2, p53, Beclin-2, and LC-3 protein expression (Figure 6) was evaluated to assess the influence of SiQDs on various cell signaling pathways involved in the response of lung and spleen cells to oxidative stress. The Nrf-2 protein is an essential transcription factor in response to antioxidants, being an indicator of oxidative stress in redox homeostasis. The increase in the level of free radicals and the presence of oxidative stress can lead to the translocation of Nrf-2 from the cytoplasm to the nucleus to initiate the antioxidant response, thereby protecting tissues against oxidative damage. The level of Nrf-2 protein decreased by 19% compared to the control in the case of lung samples at 24 h, followed by a recovery at 72 h (Figure 6b). In the case of the spleen, the level of Nrf-2 protein slightly increased by 9.4% compared to the control after 72 h, suggesting the activation of Nrf-2 signaling pathways as a defense mechanism against oxidative stress exerted by the presence of SiQDs at this level (Figure 6c).

The p53 protein has an important role in regulating the cell cycle and cell death initiation [31]. The most important change in lung tissue was the increase by 21% over the control after 72 h (Figure 6d). A similar increase in this protein level was observed in spleen tissue after 1 h, indicating the presence of an apoptotic response mediated by p53 following exposure to 100 mg SiQDs/kg b.w. After 6 and 24 h, the p53 expression in the spleen decreased compared to the control, but after 72 h, the level returned to values close to those of the control, indicating the superior ability of the spleen to neutralize the effects produced by exposure to SiQDs (Figure 6e).

The quantification of Beclin-1 and LC3 protein expression (Figure 6f–i) was performed to assess the ability of SiQDs to induce autophagy in mouse lung and spleen tissues after exposure to 100 mg SiQDs/kg b.w. Beclin-1 is a known autophagy regulator, playing a critical role in autophagosome biogenesis, regulating endocytic trafficking and LC-3-associated phagocytosis. These functions are mainly achieved by the association of Beclin-1 with other proteins and the formation of complexes responsible for phosphatidylinositol phosphorylation that mediate autophagy and transmembrane transport [32,33]. In the case of lung tissue, a 37% increase in Beclin-1 expression was observed after 1 h post injection, but after 72 h, the protein expression level returned to that of the control (Figure 6f). There were no significant changes in Beclin-1 expression in splenic tissues compared to the control throughout the 72 h experiment (Figure 6g). In addition, LC-3 plays an important role in the formation of autophagosomes and in the regulation of autophagy by recycling conjugated compounds [34]. In the case of lung tissue, a decrease by 22% of the control was observed after 24 h, but the LC3 level recovered to that of the control after 72 h (Figure 6h). Regarding the time dynamics of LC-3 protein expression in the spleen, we observed a progressive, time-dependent increase, ranging from 5% after 1 h to 19% at 72 h compared to the control (Figure 6i).

### 3.4. Genotoxicity Evaluation

#### 3.4.1. Analysis of 8-OHdG Level

In the context of DNA degradation assessment, the 8-OHdG level has to be quantified, being correlated with the oxidative stress induced by ROS [35], with important consequences such as DNA damage and cell death after exposure to nanoparticles [36]. According to our data, the level of 8-OHdG decreased by 6% and 8% below the control in the lungs and spleen, respectively, of mice exposed to 100 mg SiQD/kg b.w., indicating the efficiency of the DNA molecule repair systems, which manages to repair the possible oxidative damage produced by the presence of SiQDs.

**Figure 7:**
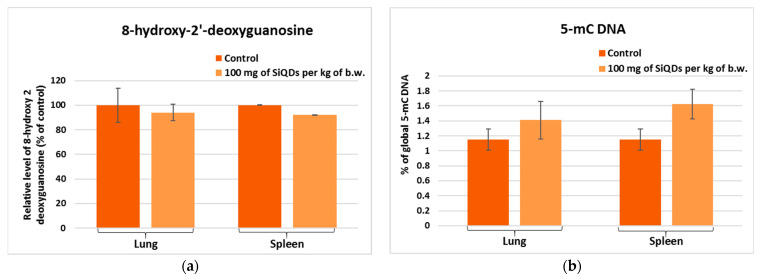
The levels of 8-OHdG (**a**) and global DNA methylation (**b**) determined by ELISA technique in the murine lung and spleen samples collected at 72 h after the administration of SiQDs (100 mg QDs/kg b.w.). Results are expressed as mean ± SD (*n* = 5).

DNA methylation is crucial in maintaining genome stability; however, it is susceptible to alterations prompted by various environmental factors. Global DNA methylation can be detected by measuring the global level of the 5-mC in the control and QD-exposed samples using an immunochemical detection method [37,38]. According to our data, the percentage of 5-mC DNA in mice exposed to 100 mg SiQD/kg b.w. increased by 22% and 41% above that of the control in lung and spleen samples, respectively (Figure 7b), confirming cytosine methylation at the DNA level.

#### 3.4.2. Analysis of Histone H4 Modification

**Figure 8:**
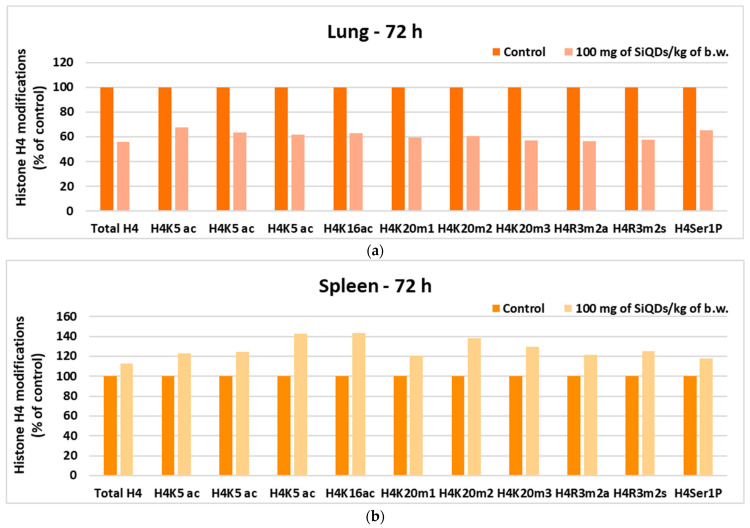
Changes in histone H4 in lung (**a**) and spleen (**b**) samples collected 72 h after SiQD administration (100 mg QDs/kg b.w.).

The analysis of histone H4 changes in lung tissue indicated a level of H4 methylation below 65% of that of the control for all tested variants (Figure 8a), while in spleen tissue, an increase in the degree of histone methylation was observed for all tested variants, with the level of methylation being higher than 120% of that of the control (Figure 8b), suggesting a superior sensitivity of the spleen to the toxic effects of SiQDs, because it is one of the main organs that accumulate QDs, also representing the site of immune response generation.

## 4. Discussion

In the present study, the toxic effects triggered by SiQDs on mice lungs and spleen were investigated in order to analyze the mechanisms by which these QDs can induce toxicity after an in vivo exposure at concentrations ranging from 1 to 100 mg SiQD/kg b.w. for a period of up to 72 h post administration. For this purpose, tissue morphology, oxidative stress, the expression of apoptosis and autophagy proteins, and DNA damage were assessed.

The administration of SiQDs in the tail vein most likely generated their distribution into the general blood circulation. Probably due to the presence of silica shells (Figure 1b), these QDs adhered to the red blood cells (RBCs), generating a high accumulation of RBC-bound SiQDs especially in the lungs up to 6 h (Figure 2) and in the spleen, as it was previously observed [39]. Also, it is worth mentioning that the spleen and liver are natural filtration sites of QDs present in the blood, due to their tendency to filter the blood-borne molecules [40].

Free radicals are the basis of the mechanisms involved in the oxidative stress generated by exposure to SiQDs. Taking into account that SiQDs are able to interact with the lung cell membrane [19], this process can facilitate an increase in the activity of membrane NADPH oxidase (NOX), generating more superoxide anions with high cytotoxicity [41]. Moreover, once internalized by the lung cells, SiQDs could also damage mitochondrial membranes, favoring the release of superoxide anions into the cytoplasm [42]. If ROS are not counteracted by the enzymatic and non-enzymatic antioxidant systems, the oxidation of DNA, proteins, and lipids occur.

Two important types of superoxide dismutase exist in mammalian cells: the cytoplasmatic Cu,Zn-SOD and the mitochondrial Mn-SOD. Both catalyze the same reaction, i.e., the dismutation of superoxide in the presence of two protons with the generation of hydrogen peroxide, with the last one being able to inhibit Cu,Zn SOD [43]. The dose-dependent decrease in total SOD activity in lung samples (Figure 4a) could be explained by this kind of inhibition. However, although the downward trend was maintained in the 72 h samples, the SOD activity returned slightly to values similar to those of the control. A previous report by Wang et al. [21] indicated the lack of significant changes in lung SOD activity after 14 days from the administration of arginine–glycine–aspartic acid peptide-conjugated CdSeTe/ZnS QDs (NIR QDs-RGD) to mice, which might suggest that the recovery of SOD activity could be possible in a larger unit of time.

The significant decrease in CAT activity (Figure 4c) indicates a large amount of hydrogen peroxide in the lung cells, that inhibited this enzyme [44]. Hydrogen peroxide can inhibit CAT through substrate inhibition at high concentrations, leading to the accumulation of less active or inactive enzyme forms (Compounds II and III), and through oxidative damage that affects enzyme structure and activity. Taking into account that GPx has a lower K_M_ value for hydrogen peroxide compared to CAT, high levels of this substrate cannot be decomposed by GPx. The analysis of GPx activity in the lung (Figure 4g) showed a significant decrease up to 72 h, indicating that this enzyme was not efficient in removing either hydrogen or lipid peroxides by conjugating with GSH [45].

Pulmonary GST activity (Figure 4i) did not change after 1 h regardless of the dose used, and it showed a decrease in a dose-dependent manner after 6 h. The diminished GPx activities correlated with those of GST and could indicate the inability of the lung to neutralize the toxic effects produced by SiQDs.

The transient increase in Gred activity (Figure 4e) at the pulmonary level after 6 h post administration confirmed the presence of oxidative stress at this organ, as this enzyme catalyzes the reaction of the reduction of glutathione disulfide (GSSG) to GSH [46], an essential molecule in counteracting the oxidative stress. In our study, the slight decrease after the first hours followed by an increase at 6 h compared to the control correlated with the increased activity of Gred. Later on, its lung concentration was close to that of the control independent of dose and time. A similar variation in GSH concentration was produced by other types of QDs at the lung level [21].

A transient increase in lung MDA level (Figure 5c) was observed after 1 h post injection, indicating a susceptibility of this organ to lipid peroxidation, consistent with the decreased CAT and GPX activities. However, the lung successfully neutralized the toxicity after 24 h, and the MDA level returned to the control one, possibly due to the cooperation between Gred and GST activities that succeeded to remove the excess of MDA [21].

Generally, it was shown that the splenic delivery of nanoparticles is inversely proportional to the hepatic uptake due to the existence of a difference in blood flow between the spleen and liver [47] and, consequently, the toxic effects of QDs appeared later compared to the lungs.

The splenic SOD activity (Figure 4b) was almost unchanged compared to the control, except after 1 h post injection for the highest dose of SiQDs, indicating the lack of superoxide anion generation inside the splenic cells. Similar results have been obtained with other types of QDs, such as NIR QDs-RGD [21]. Instead, another study revealed that CdSe QDs induced a dose-dependent reduction in SOD activity in the rat spleen starting with a dose of 10 mg per kg of b.w. [48].

The significant decrease in the specific activity of CAT at 6 h post injection (Figure 4d) confirmed a possible direct interaction between hydrophobic residues of CAT and SiQDs, generating a change in the α-helix structure of protein as it was previously proven [49], with consequences of the decrease in enzymatic activity. However, after 24 h, the CAT activity returned to values close to those of the control, suggesting the dynamic tertiary structure of CAT, that could be re-arranged in time, restoring the enzymatic activity [50]. Similar results regarding splenic CAT activity after the administration of CdSe QDs were also reported by Das et al. [48].

The decrease in GPx activity in spleen samples (Figure 4h), recorded for all time intervals and doses, was lower compared to that measured in the lung, whereas that of GST activity was higher. Taking into account that the MDA level increased after 6 and 24 h post injection for the highest dose tested, we could consider that the GST contribution to the detoxification process of lipid peroxides was more important in spleen tissue compared to the lung.

Splenic Gred activity did not show important decreases compared to the control between 6 and 72 h after administration, and the amount of GSH was almost at the same level as the control in all groups. Possibly, significant decreases in splenic GPx and GST activities could occur despite the normal GSH level, since these QDs surrounded by protein coronas [51] could affect the tridimensional conformation of GPX and GST proteins, altering their function [52].

Redox signaling pathways present at the cellular level can be modulated by exposure to SiQDs, which amplifies the cells’ response to oxidative stress. The decrease in Nrf-2 protein expression in mice lung after 24 h from the administration of 100 mg SiQDs/kg b.w. suggested the induction of oxidative stress at this level due to the fact that this protein activates the transcription of several genes encoding antioxidant enzymes [53]. As a result, the decrease in SOD, CAT, and GPX activities could be due to both enzymatic and biosynthesis inhibition. Also, SiQDs could partially inhibit Nrf-2 synthesis in lung cells at 24 h after the administration of 100 mg SiQDs/kg b.w. As demonstrated in previous studies, the decrease in the expression of this protein underlines a disturbance in the regulation of redox homeostasis at the lung level in the presence of QDs, when the restoration of GSH homeostasis by Nrf-2 is critical for cell survival during oxidative stress [54]. Despite the transient increase in Beclin-1 level (Figure 6f) after the first hour, as a consequence of the first contact to exogenous SiQDs, the lack of further significant changes in the relative expression of Beclin-1 and LC-3 proteins indicated that the autophagic processes were not fully activated at the lung level.

Moreover, a slight increase in p53 expression was observed after 72 h in lung tissue (Figure 6d), which indicated apoptosis and correlated with the morphological changes, such as the accumulation of alveolar macrophages, depicted by histological staining in Figure 2. These findings could suggest a small degree of pulmonary fibrosis induced by 100 mg SiQDs/kg b.w. This relationship was also previously described by Zaafan at al. [55] and Wang et al. [56].

In the case of splenic tissue, no significant variation in Nrf-2 expression occurred. In contrast, the increase in the level of LC-3 at the splenic level might suggest the reduction in autophagic processes, with the SiQDs being undigested and visible in the cells (Figure 3). The decrease in p53 protein expression in splenic cells could be correlated with the increased in global DNA methylation (Figure 7b), because p53 helps maintain DNA methylation homeostasis [57]. Carbon 5 of cytosine residues can be covalently methylated after exposure to various exogenous compounds, including QDs. In addition, a slight increase in 5-mC level was observed in lung tissue, which is in agreement with similar findings noticed in vitro in NL20 lung cells exposed to titanium dioxide nanoparticles [58].

Although no significant changes compared to the control were observed for 8-OHdG (Figure 7a), there were important modifications in the case of histone H4. This can undergo covalent modifications, such as methylation, acetylation, and phosphorylation, which influence gene regulation, DNA repair mechanisms, and chromatin condensation [59]. Our results showed a decrease in histone H4 level in lung tissue as a consequence of the toxicity induced by the highest dose of SiQDs (Figure 8a), with similar results being previously reported for the same QDs within the mice liver [60]. Furthermore, the decrease in H4 level in spleen tissue compared to the control was also evidenced after exposure to zinc oxide nanoparticles [61].

Given the nature of SiQDs with a silicon/silica core–shell structure and their propensity for aggregation, their excretion route may differ from soluble forms of silicon. It is crucial to consider factors such as particle size, surface properties, and aggregation state, as these can influence the excretion kinetics. While soluble silicon typically excretes as silicic acid via urine, the larger and aggregated SiQDs may undergo different clearance mechanisms. The increased hydrodynamic diameter, higher than 15 nm, prevented the renal excretion of nanoparticles, as it was previously reported [62,63]. Other pathways of clearance might include phagocytosis by macrophages in the lungs and subsequent transport to the lymphatic system for clearance from the body, clearance via the hepatobiliary route, or accumulation in the spleen, where they could interact with immune cells or undergo degradation processes.

## 5. Conclusions

This research provided a detailed evaluation of the in vivo effect of SiQDs synthesized by laser ablation on oxidative stress, cell death, and epigenetic changes. We found that their main effect in the lung and spleen was the generation of oxidative stress, observed at doses higher than 10 mg QD/kg b.w., which included decreased CAT and GPx activities in the lung, and increased lipid peroxidation and LC3-B expression in the spleen. Also, administering a high dose of SiQDs caused alveolar macrophage accumulation and increased internalization in splenic cells. However, no significant genotoxicity was found in the analyzed tissues. In conclusion, our results provide valuable information for future clinical trials, indicating that doses up to 10 mg SiQD/kg bw. did not induce pulmonary and splenic toxicity. However, further studies, including biodistribution and excretion studies, are warranted to elucidate the specific excretion pathways of SiQDs.

## Data Availability

The raw data supporting the conclusions of this article will be made available by the authors on request.
