# Correction: Cristian et al. Exploring In Vivo Pulmonary and Splenic Toxicity Profiles of Silicon Quantum Dots in Mice. *Materials* 2024, *17*, 2778

**DOI:** 10.3390/ma19010141

**Published:** 2025-12-31

**Authors:** Roxana-Elena Cristian, Cornel Balta, Hildegard Herman, Alina Ciceu, Bogdan Trica, Beatrice G. Sbarcea, Eftimie Miutescu, Anca Hermenean, Anca Dinischiotu, Miruna S. Stan

**Affiliations:** 1 Department of Biochemistry and Molecular Biology, Faculty of Biology, University of Bucharest, 91-95 Splaiul Independentei, 050095 Bucharest, Romania; roxana.cristian@drd.unibuc.ro (R.-E.C.); anca.hermenean@gmail.com (A.H.); miruna.stan@bio.unibuc.ro (M.S.S.); 2 DANUBIUS Department, National Institute of Research and Development for Biological Sciences, Splaiul Independentei 296, 060031 Bucharest, Romania; 3 “Aurel Ardelean” Institute of Life Sciences, Vasile Goldis Western University of Arad, 86 Rebreanu, 310414 Arad, Romania; balta.cornel@uvvg.ro (C.B.); herman.hildegard@uvvg.ro (H.H.); alinaciceu80@gmail.com (A.C.); 4 National Institute for Research & Development in Chemistry and Petrochemistry (INCDCP-ICECHIM), 202 Spl. Independentei, 060021 Bucharest, Romania; trica.bogdan@gmail.com; 5 Materials Characterization Department, National Institute for Research & Development in Electrical Engineering (ICPE-CA), 313 Splaiul Unirii, 030138 Bucharest, Romania; gabriela.sbarcea@icpe-ca.ro; 6 Faculty of Medicine, Vasile Goldis Western University of Arad, 86 Rebreanu, 310414 Arad, Romania; miutescu.eftimie@uvvg.ro; 7 Research Institute of the University of Bucharest (ICUB), University of Bucharest, 91-95 Spl. Independentei, 050095 Bucharest, Romania

In the original publication [1], there was an overlap in Figure 2 as published. The corrected Figure 2 appears below. The authors state that the scientific conclusions are unaffected. This correction was approved by the Academic Editor. The original publication has also been updated.

**Figure 2:**
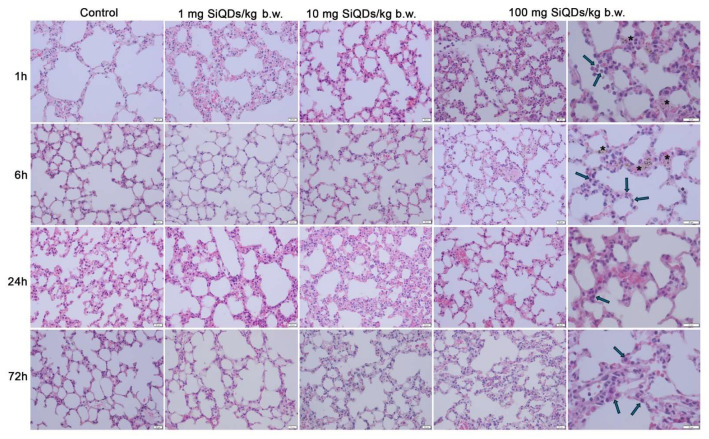
Histopathological evaluation of lung tissue using hematoxylin and eosin staining at intervals of 1, 6, 24, and 72 h post administration of 1 mg, 10 mg, and 100 mg of SiQDs/kg of b.w. Legend: arrows—alveolar macrophages; black stars—SiQD accumulation (brown). Scale bar (represented in the lower right corner of each image): 20 μm.
